# 49156 Effects of Race and Demographics on Use of Physical Restraints in the Emergency Department

**DOI:** 10.1017/cts.2021.710

**Published:** 2021-03-30

**Authors:** Ambrose H. Wong, Travis Whitfill, Emmanuel C. Ohuabunwa, Jessica M. Ray, James D. Dziura, Steven L. Bernstein, Richard Andrew Taylor

**Affiliations:** Department of Emergency Medicine, Yale School of Medicine

## Abstract

ABSTRACT IMPACT: Within three EDs in a regional health system in Connecticut, African American race, male gender, non-Hispanic ethnicity, lack of private insurance, and homelessness were associated with significant odds of being physically restrained during a visit. OBJECTIVES/GOALS: Agitated patient encounters in the Emergency Department (ED) are on the rise, and physical restraints are used to protect staff and prevent self-harm. However, these are associated with safety risks and potential stigmatization of vulnerable individuals. We aim to determine factors that are associated with odds of being restrained in the ED. METHODS/STUDY POPULATION: We conducted a retrospective cohort analysis of all patients (≥18 yo) placed in restraints during an ED visit to three hospitals within a large tertiary health system from Jan 2013-Aug 2018. We undertook descriptive analysis of the data and created a generalized linear mixed model with a binary logistic identity link to model restraint use and determine odds ratios for various clinically significant demographic factors. These include gender, race, ethnicity, insurance status, alcohol use, illicit drug use, and homelessness. Our model accounted for patients nested across the three EDs and also accounted for multiple patient visits. RESULTS/ANTICIPATED RESULTS: In 726,417 total ED visits, 7,090 (1%) had associated restraint orders. Restrained patients had an average age of 45, with 64% male, 54% Caucasian and 29% African American. 17% had private insurance, 36% endorsed illicit substances, 51.4% endorsed alcohol use and 2.3% were homeless. African Americans had statistically significant odds of being restrained compared to Caucasians with adjusted odds ratio (AOR) of 1.14 (1.08,1.21). Females (AOR 0.75 [0.71, 0.79] had lower odds of being restrained compared to males while patients with Medicaid (AOR 1.57 [1.46, 1.68]) and Medicare (AOR 1.70 [1.57, 1.85]) had increased odds compared to the privately insured. Illicit substance use (AOR 1.55 [1.46, 1.64]), alcohol use (AOR 1.13 [1.07, 1.20] and homelessness (AOR 1.35 [1.14, 1.16]) had increased odds of restraint use. DISCUSSION/SIGNIFICANCE OF FINDINGS: We showed statistically significant effects of patient demographics on odds of restraint use in the ED. The increased odds based on race, insurance status, and substance use highlight the potential effects of implicit bias on the decision to physically restrain patients and underscores the importance of objective assessments of these patients.

